# Supplementary material for: Iterative improvement in the automatic modular design of robot swarms
Source: PeerJ Comput Sci. 2020 Dec 7;6:e322. doi: 10.7717/peerj-cs.322 (PMC7924708; doi:10.7717/peerj-cs.322)
Supplement: Supplemental Information 3 [file peerj-cs-06-322-s003.zip › argos3/doc/api/standalone/a00556_source.html]

ARGoS: plugins/robots/mini-quadrotor/simulator/qtopengl\_miniquadrotor.cpp Source File


- Main Page
- Related Pages
- Namespaces
- Classes
- Files

- File List
- File Members

# plugins/robots/mini-quadrotor/simulator/qtopengl\_miniquadrotor.cpp

Go to the documentation of this file.

```
00001 
00007 #include "qtopengl_miniquadrotor.h"
00008 #include "miniquadrotor_entity.h"
00009 #include <argos3/core/utility/math/vector2.h>
00010 #include <argos3/core/utility/math/vector3.h>
00011 #include <argos3/plugins/simulator/visualizations/qt-opengl/qtopengl_widget.h>
00012 
00013 namespace argos {
00014 
00015    /****************************************/
00016    /****************************************/
00017 
00018    /* All measures are in meters */
00019 
00020    static const Real BOARD_HALF_DIAGONAL    = 0.029f;
00021    static const Real BOARD_HEIGHT           = 0.005f;
00022 
00023    static const Real ARM_HALF_LENGTH        = 0.063f;
00024    static const Real ARM_HEIGHT             = 0.003f;
00025    static const Real ARM_HALF_WIDTH         = 0.0015f;
00026    static const Real ARM_BOTTOM             = 0.001f;
00027    static const Real ARM_TOP                = ARM_BOTTOM + ARM_HEIGHT;
00028 
00029    static const Real PROPELLER_ROD_RADIUS   = 0.013f;
00030    static const Real PROPELLER_ROD_HEIGHT   = 0.011f;
00031    static const Real PROPELLER_ROD_BOTTOM   = ARM_TOP;
00032    static const Real PROPELLER_ROD_TOP      = PROPELLER_ROD_BOTTOM + PROPELLER_ROD_HEIGHT;
00033 
00034    static const Real PROPELLER_BLADE_RADIUS    = 0.041f;
00035    static const Real PROPELLER_BLADE_ELEVATION = 0.0105f;
00036 
00037    static const Real ONE_OVER_SR2           = 1.0f / Sqrt(2.0f);
00038 
00039    /****************************************/
00040    /****************************************/
00041 
00042    CQTOpenGLMiniQuadrotor::CQTOpenGLMiniQuadrotor() :
00043       m_unVertices(40) {
00044       /* Reserve the needed display lists */
00045       m_unLists = glGenLists(1);
00046       /* Create the body display list */
00047       glNewList(m_unLists, GL_COMPILE);
00048       MakeBody();
00049       glEndList();
00050    }
00051 
00052    /****************************************/
00053    /****************************************/
00054 
00055    CQTOpenGLMiniQuadrotor::~CQTOpenGLMiniQuadrotor() {
00056       glDeleteLists(m_unLists, 1);
00057    }
00058 
00059    /****************************************/
00060    /****************************************/
00061 
00062    void CQTOpenGLMiniQuadrotor::Draw(CMiniQuadrotorEntity& c_entity) {
00063       /* Place the body */
00064       glCallList(m_unLists);
00065    }
00066 
00067    /****************************************/
00068    /****************************************/
00069 
00070    void CQTOpenGLMiniQuadrotor::SetBodyMaterial() {
00071       const GLfloat pfColor[]     = {   0.00f, 0.00f, 0.00f, 1.00f };
00072       const GLfloat pfSpecular[]  = {   0.90f, 0.90f, 0.90f, 1.00f };
00073       const GLfloat pfShininess[] = { 100.00f                   };
00074       const GLfloat pfEmission[]  = {   0.00f, 0.00f, 0.00f, 1.00f };
00075       glMaterialfv(GL_FRONT_AND_BACK, GL_AMBIENT_AND_DIFFUSE, pfColor);
00076       glMaterialfv(GL_FRONT_AND_BACK, GL_SPECULAR,            pfSpecular);
00077       glMaterialfv(GL_FRONT_AND_BACK, GL_SHININESS,           pfShininess);
00078       glMaterialfv(GL_FRONT_AND_BACK, GL_EMISSION,            pfEmission);
00079    }
00080 
00081    /****************************************/
00082    /****************************************/
00083 
00084    void CQTOpenGLMiniQuadrotor::SetBoardMaterial() {
00085       const GLfloat pfColor[]     = {   0.00f, 1.00f, 0.00f, 1.00f };
00086       const GLfloat pfSpecular[]  = {   0.90f, 0.90f, 0.90f, 1.00f };
00087       const GLfloat pfShininess[] = { 100.00f                      };
00088       const GLfloat pfEmission[]  = {   0.00f, 0.00f, 0.00f, 1.00f };
00089       glMaterialfv(GL_FRONT_AND_BACK, GL_AMBIENT_AND_DIFFUSE, pfColor);
00090       glMaterialfv(GL_FRONT_AND_BACK, GL_SPECULAR,            pfSpecular);
00091       glMaterialfv(GL_FRONT_AND_BACK, GL_SHININESS,           pfShininess);
00092       glMaterialfv(GL_FRONT_AND_BACK, GL_EMISSION,            pfEmission);
00093    }
00094 
00095    /****************************************/
00096    /****************************************/
00097 
00098    void CQTOpenGLMiniQuadrotor::SetBladeMaterial() {
00099       const GLfloat pfColor[]     = {   0.75f, 0.75f, 0.75f, 1.00f };
00100       const GLfloat pfSpecular[]  = {   0.90f, 0.90f, 0.90f, 1.00f };
00101       const GLfloat pfShininess[] = { 100.00f                   };
00102       const GLfloat pfEmission[]  = {   0.00f, 0.00f, 0.00f, 1.00f };
00103       glMaterialfv(GL_FRONT_AND_BACK, GL_AMBIENT_AND_DIFFUSE, pfColor);
00104       glMaterialfv(GL_FRONT_AND_BACK, GL_SPECULAR,            pfSpecular);
00105       glMaterialfv(GL_FRONT_AND_BACK, GL_SHININESS,           pfShininess);
00106       glMaterialfv(GL_FRONT_AND_BACK, GL_EMISSION,            pfEmission);
00107    }
00108 
00109    /****************************************/
00110    /****************************************/
00111 
00112    void CQTOpenGLMiniQuadrotor::MakeBody() {
00113       /* Board */
00114       MakeBoard();
00115       /* N->S arm */
00116       MakeArm();
00117       /* W->E arm */
00118       glPushMatrix();
00119       glRotatef(90.0f, 0.0f, 0.0f, 1.0f);
00120       MakeArm();
00121       glPopMatrix();
00122       /* Propellers */
00123       /* N propeller */
00124       glPushMatrix();
00125       glTranslatef(0.0f, ARM_HALF_LENGTH - PROPELLER_ROD_RADIUS, 0.0f);
00126       MakePropeller();
00127       glPopMatrix();
00128       /* E propeller */
00129       glPushMatrix();
00130       glTranslatef(ARM_HALF_LENGTH - PROPELLER_ROD_RADIUS, 0.0f, 0.0f);
00131       MakePropeller();
00132       glPopMatrix();
00133       /* S propeller */
00134       glPushMatrix();
00135       glTranslatef(0.0f, -ARM_HALF_LENGTH + PROPELLER_ROD_RADIUS, 0.0f);
00136       MakePropeller();
00137       glPopMatrix();
00138       /* W propeller */
00139       glPushMatrix();
00140       glTranslatef(-ARM_HALF_LENGTH + PROPELLER_ROD_RADIUS, 0.0f, 0.0f);
00141       MakePropeller();
00142       glPopMatrix();
00143    }
00144 
00145    /****************************************/
00146    /****************************************/
00147 
00148    void CQTOpenGLMiniQuadrotor::MakeBoard() {
00149       SetBoardMaterial();
00150       glBegin(GL_QUADS);
00151       /* Bottom face, parallel to XY */
00152       glNormal3f(0.0f, 0.0f, -1.0f);
00153       glVertex3f(                0.0f,  BOARD_HALF_DIAGONAL, 0.0f        );
00154       glVertex3f( BOARD_HALF_DIAGONAL,                 0.0f, 0.0f        );
00155       glVertex3f(                0.0f, -BOARD_HALF_DIAGONAL, 0.0f        );
00156       glVertex3f(-BOARD_HALF_DIAGONAL,                 0.0f, 0.0f        );
00157       /* Top face, parallel to XY */
00158       glNormal3f(0.0f, 0.0f, 1.0f);
00159       glVertex3f(                0.0f,  BOARD_HALF_DIAGONAL, BOARD_HEIGHT);
00160       glVertex3f(-BOARD_HALF_DIAGONAL,                 0.0f, BOARD_HEIGHT);
00161       glVertex3f(                0.0f, -BOARD_HALF_DIAGONAL, BOARD_HEIGHT);
00162       glVertex3f( BOARD_HALF_DIAGONAL,                 0.0f, BOARD_HEIGHT);
00163       /* This part covers the faces (NE, SE, SW, NW) */
00164       /* NE face */
00165       glNormal3f(        ONE_OVER_SR2,         ONE_OVER_SR2, 0.0f        );
00166       glVertex3f(                0.0f,  BOARD_HALF_DIAGONAL, 0.0f        );
00167       glVertex3f(                0.0f,  BOARD_HALF_DIAGONAL, BOARD_HEIGHT);
00168       glVertex3f( BOARD_HALF_DIAGONAL,                 0.0f, BOARD_HEIGHT);
00169       glVertex3f( BOARD_HALF_DIAGONAL,                 0.0f, 0.0f        );
00170       /* SE face */
00171       glNormal3f(        ONE_OVER_SR2,        -ONE_OVER_SR2, 0.0f        );
00172       glVertex3f( BOARD_HALF_DIAGONAL,                 0.0f, 0.0f        );
00173       glVertex3f( BOARD_HALF_DIAGONAL,                 0.0f, BOARD_HEIGHT);
00174       glVertex3f(                0.0f, -BOARD_HALF_DIAGONAL, BOARD_HEIGHT);
00175       glVertex3f(                0.0f, -BOARD_HALF_DIAGONAL, 0.0f        );
00176       /* SW face */
00177       glNormal3f(       -ONE_OVER_SR2,        -ONE_OVER_SR2, 0.0f        );
00178       glVertex3f(                0.0f, -BOARD_HALF_DIAGONAL, 0.0f        );
00179       glVertex3f(                0.0f, -BOARD_HALF_DIAGONAL, BOARD_HEIGHT);
00180       glVertex3f(-BOARD_HALF_DIAGONAL,                 0.0f, BOARD_HEIGHT);
00181       glVertex3f(-BOARD_HALF_DIAGONAL,                 0.0f, 0.0f        );
00182       /* NW face */
00183       glNormal3f(       -ONE_OVER_SR2,         ONE_OVER_SR2, 0.0f        );
00184       glVertex3f(-BOARD_HALF_DIAGONAL,                 0.0f, 0.0f        );
00185       glVertex3f(-BOARD_HALF_DIAGONAL,                 0.0f, BOARD_HEIGHT);
00186       glVertex3f(                0.0f,  BOARD_HALF_DIAGONAL, BOARD_HEIGHT);
00187       glVertex3f(                0.0f,  BOARD_HALF_DIAGONAL, 0.0f        );
00188       glEnd();
00189    }
00190 
00191    /****************************************/
00192    /****************************************/
00193 
00194    void CQTOpenGLMiniQuadrotor::MakeArm() {
00195       SetBodyMaterial();
00196       glBegin(GL_QUADS);
00197       /* Bottom face, parallel to XY */
00198       glNormal3f( 0.0f,            0.0f,            -1.0f      );
00199       glVertex3f( ARM_HALF_WIDTH,  ARM_HALF_LENGTH,  ARM_BOTTOM);
00200       glVertex3f( ARM_HALF_WIDTH, -ARM_HALF_LENGTH,  ARM_BOTTOM);
00201       glVertex3f(-ARM_HALF_WIDTH, -ARM_HALF_LENGTH,  ARM_BOTTOM);
00202       glVertex3f(-ARM_HALF_WIDTH,  ARM_HALF_LENGTH,  ARM_BOTTOM);
00203       /* Top face, parallel to XY */
00204       glNormal3f( 0.0f,            0.0f,             1.0f      );
00205       glVertex3f( ARM_HALF_WIDTH,  ARM_HALF_LENGTH,  ARM_TOP   );
00206       glVertex3f(-ARM_HALF_WIDTH,  ARM_HALF_LENGTH,  ARM_TOP   );
00207       glVertex3f(-ARM_HALF_WIDTH, -ARM_HALF_LENGTH,  ARM_TOP   );
00208       glVertex3f( ARM_HALF_WIDTH, -ARM_HALF_LENGTH,  ARM_TOP   );
00209       /* This part covers the faces (N, E, S, W) */
00210       /* North face */
00211       glNormal3f( 1.0f,            0.0f,             0.0f      );
00212       glVertex3f( ARM_HALF_WIDTH,  ARM_HALF_LENGTH,  ARM_TOP   );
00213       glVertex3f( ARM_HALF_WIDTH,  ARM_HALF_LENGTH,  ARM_BOTTOM);
00214       glVertex3f(-ARM_HALF_WIDTH,  ARM_HALF_LENGTH,  ARM_BOTTOM);
00215       glVertex3f(-ARM_HALF_WIDTH,  ARM_HALF_LENGTH,  ARM_TOP   );
00216       /* East face */
00217       glNormal3f( 0.0f,            1.0f,             0.0f      );
00218       glVertex3f( ARM_HALF_WIDTH,  ARM_HALF_LENGTH,  ARM_TOP   );
00219       glVertex3f( ARM_HALF_WIDTH, -ARM_HALF_LENGTH,  ARM_TOP   );
00220       glVertex3f( ARM_HALF_WIDTH, -ARM_HALF_LENGTH,  ARM_BOTTOM);
00221       glVertex3f( ARM_HALF_WIDTH,  ARM_HALF_LENGTH,  ARM_BOTTOM);
00222       /* South face */
00223       glNormal3f(-1.0f,            0.0f,             0.0f      );
00224       glVertex3f( ARM_HALF_WIDTH, -ARM_HALF_LENGTH,  ARM_TOP   );
00225       glVertex3f(-ARM_HALF_WIDTH, -ARM_HALF_LENGTH,  ARM_TOP   );
00226       glVertex3f(-ARM_HALF_WIDTH, -ARM_HALF_LENGTH,  ARM_BOTTOM);
00227       glVertex3f( ARM_HALF_WIDTH, -ARM_HALF_LENGTH,  ARM_BOTTOM);
00228       /* West face */
00229       glNormal3f( 0.0f,           -1.0f,             0.0f      );
00230       glVertex3f(-ARM_HALF_WIDTH,  ARM_HALF_LENGTH,  ARM_TOP   );
00231       glVertex3f(-ARM_HALF_WIDTH,  ARM_HALF_LENGTH,  ARM_BOTTOM);
00232       glVertex3f(-ARM_HALF_WIDTH, -ARM_HALF_LENGTH,  ARM_BOTTOM);
00233       glVertex3f(-ARM_HALF_WIDTH, -ARM_HALF_LENGTH,  ARM_TOP   );
00234       glEnd();      
00235    }
00236 
00237    /****************************************/
00238    /****************************************/
00239 
00240    void CQTOpenGLMiniQuadrotor::MakePropeller() {
00241       SetBodyMaterial();
00242       /* Rod side surface */
00243       CVector2 cNormal(1.0f, 0.0f);
00244       CVector2 cVertex;
00245       CRadians cAngle(CRadians::TWO_PI / m_unVertices);
00246       glBegin(GL_QUAD_STRIP);
00247       for(GLuint i = 0; i <= m_unVertices; i++) {
00248          glNormal3f(cNormal.GetX(), cNormal.GetY(), 0.0f);
00249          cVertex = cNormal;
00250          cVertex *= PROPELLER_ROD_RADIUS;
00251          glVertex3f(cVertex.GetX(), cVertex.GetY(), PROPELLER_ROD_TOP);
00252          glVertex3f(cVertex.GetX(), cVertex.GetY(), PROPELLER_ROD_BOTTOM);
00253          cNormal.Rotate(cAngle);
00254       }
00255       glEnd();
00256       /* Rod top disk */
00257       cVertex.Set(PROPELLER_ROD_RADIUS, 0.0f);
00258       glBegin(GL_POLYGON);
00259       glNormal3f(0.0f, 0.0f, 1.0f);
00260       for(GLuint i = 0; i <= m_unVertices; i++) {
00261          glVertex3f(cVertex.GetX(), cVertex.GetY(), PROPELLER_ROD_TOP);
00262          cVertex.Rotate(cAngle);
00263       }
00264       glEnd();
00265       /* Rod bottom disk */
00266       cVertex.Set(PROPELLER_ROD_RADIUS, 0.0f);
00267       cAngle = -cAngle;
00268       glBegin(GL_POLYGON);
00269       glNormal3f(0.0f, 0.0f, -1.0f);
00270       for(GLuint i = 0; i <= m_unVertices; i++) {
00271          glVertex3f(cVertex.GetX(), cVertex.GetY(), PROPELLER_ROD_BOTTOM);
00272          cVertex.Rotate(cAngle);
00273       }
00274       glEnd();
00275       /* Rotating blade disks */
00276       SetBladeMaterial();
00277       cVertex.Set(PROPELLER_BLADE_RADIUS, 0.0f);
00278       cAngle = -cAngle;
00279       glDisable(GL_CULL_FACE);
00280       glBegin(GL_POLYGON);
00281       glNormal3f(0.0f, 0.0f, 1.0f);
00282       for(GLuint i = 0; i <= m_unVertices; i++) {
00283          glVertex3f(cVertex.GetX(), cVertex.GetY(), PROPELLER_BLADE_ELEVATION);
00284          cVertex.Rotate(cAngle);
00285       }
00286       glEnd();
00287       glEnable(GL_CULL_FACE);
00288    }
00289 
00290    /****************************************/
00291    /****************************************/
00292 
00293    class CQTOpenGLOperationDrawMiniQuadrotorNormal : public CQTOpenGLOperationDrawNormal {
00294    public:
00295       void ApplyTo(CQTOpenGLWidget& c_visualization,
00296                    CMiniQuadrotorEntity& c_entity) {
00297          static CQTOpenGLMiniQuadrotor m_cModel;
00298          c_visualization.DrawRays(c_entity.GetControllableEntity());
00299          c_visualization.DrawEntity(c_entity.GetEmbodiedEntity());
00300          m_cModel.Draw(c_entity);
00301       }
00302    };
00303 
00304    class CQTOpenGLOperationDrawMiniQuadrotorSelected : public CQTOpenGLOperationDrawSelected {
00305    public:
00306       void ApplyTo(CQTOpenGLWidget& c_visualization,
00307                    CMiniQuadrotorEntity& c_entity) {
00308          c_visualization.DrawBoundingBox(c_entity.GetEmbodiedEntity());
00309       }
00310    };
00311 
00312    REGISTER_QTOPENGL_ENTITY_OPERATION(CQTOpenGLOperationDrawNormal, CQTOpenGLOperationDrawMiniQuadrotorNormal, CMiniQuadrotorEntity);
00313 
00314    REGISTER_QTOPENGL_ENTITY_OPERATION(CQTOpenGLOperationDrawSelected, CQTOpenGLOperationDrawMiniQuadrotorSelected, CMiniQuadrotorEntity);
00315 
00316    /****************************************/
00317    /****************************************/
00318 
00319 }
```

---

Generated on 10 Jul 2018 for ARGoS by 
 1.6.1
